# miR-224 targets *BTRC* and promotes cell migration and invasion in colorectal cancer

**DOI:** 10.1007/s13205-020-02477-x

**Published:** 2020-10-24

**Authors:** Qi Zheng, Jane J. Yu, Chenggang Li, Jiali Li, Jiping Wang, Shuyang Wang

**Affiliations:** 1 Department of Pathology, School of Basic Medical Sciences, Fudan University, 131 Dong’an Road, Shanghai, 200032 Shanghai China; 2 Department of Internal Medicine, University of Cincinnati College of Medicine, Cincinnati, OH 45267 USA; 3 State Key Laboratory of Medical Chemical Biology and College of Pharmacy, Nankai University, Tianjin, China; 4 Department of Pathology, Huashan Hospital, Fudan University, Shanghai, China; 5 Division of Surgical Oncology, Harvard Medical School, Brigham and Women’s Hospital, Boston, MA USA

**Keywords:** miR-224, MicroRNA, Colorectal cancer, CRC, BTRC, Beta-TrCP

## Abstract

Our study aims to investigate the impact of miR-224 on cell migration and invasion in colorectal cancer (CRC) as well as its molecular mechanisms. The results showed that miR-224 was significantly upregulated in CRC compared to normal tissues via the TCGA database. Overexpression of miR-224 promoted CRC cell migration and invasion, while inhibition of miR-224 demonstrated the opposite result via transwell assays. In addition, we found that *BTRC* was a target gene of miR-224 through the miRecords database and dual-luciferase assay, while western blot together with RT-qPCR showed that inhibition of miR-224 led to elevated *BTRC* expression in protein level but not in mRNA level, and also decreased the expression of β-catenin. In reference to the Human Protein Atlas, BTRC protein expression was higher in normal tissues than in CRC tissues. In conclusion, miR-224 regulates its target BTRC protein expression and its related Wnt/β-catenin pathway. Its impact on cell migration and invasion in CRC cells suggested that miR-224 could be a prospective therapeutic target for early-stage non-metastatic CRC.

**Electronic supplementary material:**

The online version of this article (10.1007/s13205-020-02477-x) contains supplementary material, which is available to authorized users.

## Introduction

Colorectal cancer (CRC) is one of the most common malignant neoplasms in the world (Bray et al. 2018) and metastasis is one of its leading causes of death. Nevertheless, there are still few biomarkers or treatment targets for high-risk early-stage CRC patients who are prone to metastasize, and the mechanism of metastasis during the early stage of CRC is still unclear.

MicroRNAs (miRNAs) are single-strand small non-coding RNAs consisting of 19–25 nucleotides, which usually imperfectly bind to the seed regions within the 3′UTR of complementary target mRNAs resulting in translational repression or mRNA cleavage (Bartel 2004). The dysregulation of most miRNAs has been reported in a wide range of human cancer types (Iorio and Croce 2012). Congruently, many miRNAs are located in chromosomal regions that are prone to deletions or amplifications in the development of many different types of cancer (Calin et al. 2004). The amplification of those regions containing onco-miR would increase miRNA expression and silence targeted tumor suppressor genes. Conversely, miRNAs involved in the suppression of oncogenes are often located in the fragile loci where deletions or mutations tend to occur, leading to decreased miRNA levels and overexpression of the targeted oncogenes (Iorio and Croce 2012).

The aberrant expression of hsa-miR-224-5p (miR-224) has been found to differ in distinct tumor tissues in comparison to adjacent normal tissues. It has been reported that miR-224 was upregulated in pancreatic cancer (Zhu et al. 2018), renal cell carcinoma (Pan et al. 2018), cervical carcinoma (Yu et al. 2018), hepatocellular carcinoma (Hung et al. 2018), and bladder cancer (Miao et al. 2018), but low expression of miR-224 has been reported in prostate cancer (Gan et al. 2018) and oral squamous cell carcinoma (Lu et al. 2019). miR-224 seems to function differently in different cancer types. Thus, in this study, we aim to investigate the effect of miR-224 on CRC as well as its biological mechanism in HCT-116 and DLD-1 cells. Our results indicate that miR-224 level is increased in CRC tissues compared to normal tissues. Beta-Transducin Repeat Containing E3 Ubiquitin Protein Ligase *(BTRC)* is a novel target of miR-224, and miR-224 can promote cell migration and invasion of HCT-116 and DLD-1 and regulate *BTRC*-related Wnt/β-catenin pathway. Furthermore, compared to normal tissues, *BTRC* expression is reduced in CRC tissues in protein level but not in mRNA level. Our results suggest that miR-224 might be a potential biomarker or target for high-risk non-metastatic CRC patients.

## Materials and methods

### Cell culture

Human embryonic kidney cell lines (HEK293T cells) and human colorectal cancer cell lines (HCT-116 and DLD-1 cells) were purchased from the Shanghai Institute of Cell Biology, Chinese Academy of Sciences (Shanghai, China) and cultured in Dulbecco’s modified eagle’s medium (DMEM, Corning, USA) supplemented with 10% fetal bovine serum (BioInd, Israel) and 1% penicillin streptomycin (Invitrogen, USA).

### Reconstruction of the 3′UTR of *BTRC*

pmirGLO vector (Promega, USA) was digested at PmeI and XbaI and inserted with a sequence harboring the seed region in the 3′UTR of *BTRC* to reconstruct pmirGLO BTRC 3′UTR-WT (wild type), along with the corresponding mutated seed nucleotides to reconstruct pmirGLO BTRC 3′UTR-MUT (mutant) by Genewiz (China). The sequences that we inserted into pmirGLO for plasmid reconstruction plasmid are shown in Supplementary Table 1.

### miRNAs transfection and reconstructed pmirGLO co-transfection

HCT-116 and DLD-1 cells were transfected with 50 nM miR-224 mimic, 100 nM inhibitor or their corresponding negative controls (NC, RiboBio, China) in 6-well plates, respectively, using lipofectamine 2000 transfection reagent (Life technologies, USA) in opti-MEM by reference to the lipofectamine 2000 reagent protocol.

HEK293T cells were co-transfected with 20 nM miR-224 mimic or NC and 5 ng pmirGLO BTRC 3′UTR-WT or pmirGLO BTRC 3′UTR-MUT in 48-well plates, respectively, using lipofectamine 2000 in opti-MEM by reference to the *siRNA Plasmid Co*-*Transfection Protocol with Lipofectamine 2000* in its official online website.

### Cell migration and invasion assay

Cell migratory and invasive abilities were assessed using transwell inserts (8 μm, Corning, USA). HCT-116 and DLD-1 cells (around 1 × 10^5^ cells) were digested and seeded on the upper chamber of inserts after 24 h of transfection of miR-224 mimic, inhibitor or NC, respectively, in DMEM medium, while DMEM medium supplemented with 10% fetal bovine serum was placed in the lower chamber. Penetrated cells were fixed and then stained with 0.1% crystal violet and counted. For invasion assay, extracellular matrigel (BD, USA) was pre-coated in upper chamber of inserts.

### Cell RNA extraction and quantitative real-time polymerase chain reactions (qRT-PCR)

Total cell RNA was extracted using Animal tissue/Cell Total RNA Kit (Zomanbio, China). RNA concentrations were quantified by NanoDrop 1000 Spectrophotometer (NanoDrop Technologies, USA). miR-224 expression level was measured by qRT-PCR using Taqman MicroRNA Assays (Applied Biosystems, USA) with U47 expression as an endogenous control. The expression of *BTRC* mRNA was measured using Power SYBR Green PCR Master Mix (Applied Biosystems, USA) with GAPDH expression as an endogenous control. The primer sequences used for SYBR RT-qPCR are listed in Supplementary Table 2.

### Western blot analysis

Total proteins were lysed by RIPA (Beyotime, China) 48 h after the transfection. The protein expressions of the following antibodies were detected: BTRC (1:1000, A18232, Abclonal), pGSK3β-Ser9 (1:1000, 5558T, Cell Signaling Technology), β-catenin (1:4000, 51067-2-AP, Proteintech), and GAPDH (sc-166574, 1:1000, Santa Cruz Biotechnology) as an endogenous control. The gray values of western blot bands were measured by ImageJ.

### Bioinformatic analysis

The TCGA database was used for gain of miR-224 expression in CRC. Candidate targets of miR-224 were predicted by at least 4 of 11 databases in miRecords (http://c1.accurascience.com/miRecords/), and the KEGG database (http://www.kegg.jp/kegg/download/kegtools.html) was used to map the predicted candidate targets to cancer-related pathways. We obtained the data on *BTRC* transcripts in colon adenocarcinoma (COAD) and rectum adenocarcinoma (READ) from the TCGA database via GEPIA (http://gepia.cancer-pku.cn). All parameters were default.

### Dual-luciferase reporter assay

HEK293T cells (48 h after co-transfection) were processed using Dual-Luciferase Reporter Gene Assay Kit (Yeasen, China) and analyzed by GloMax^®^ 20/20 Luminometer (Promega, USA). Firefly luciferase activity was normalized to Renilla luciferase activity accordingly.

### Immunohistochemical analysis of BTRC

The immunohistochemistry (IHC) pictures of BTRC in colorectal cancer (3 cases) and normal colorectal tissues (3 cases) were obtained from the Human Protein Atlas (https://www.proteinatlas.org/). We analyzed the mean gray value of five colorectal epithelial regions, which was normalized to the mean gray value of five stromal regions in the reverted red channel of each IHC picture by ImageJ: relative gray value = (The intensity of BTRC expression in normal colorectal epithelium or colorectal tumor)/(The intensity of BTRC expression in colorectal stromal cells).

### Statistical analysis

The expressions of miR-224 from TCGA were analyzed by unpaired *t* test with Benjaminie–Hochberg correction and differences between groups in biological triplicates were analyzed by a two-tailed *t* test. IBM statistics SPSS 22 software was used for statistical analysis. All data were shown as mean ± standard deviation (SD) and *p *< 0.05 was considered as statistically significant.

## Results

### miR-224 is elevated in CRC tissues and cell lines and promotes cell migration and invasion in vitro

**Fig. 1:**
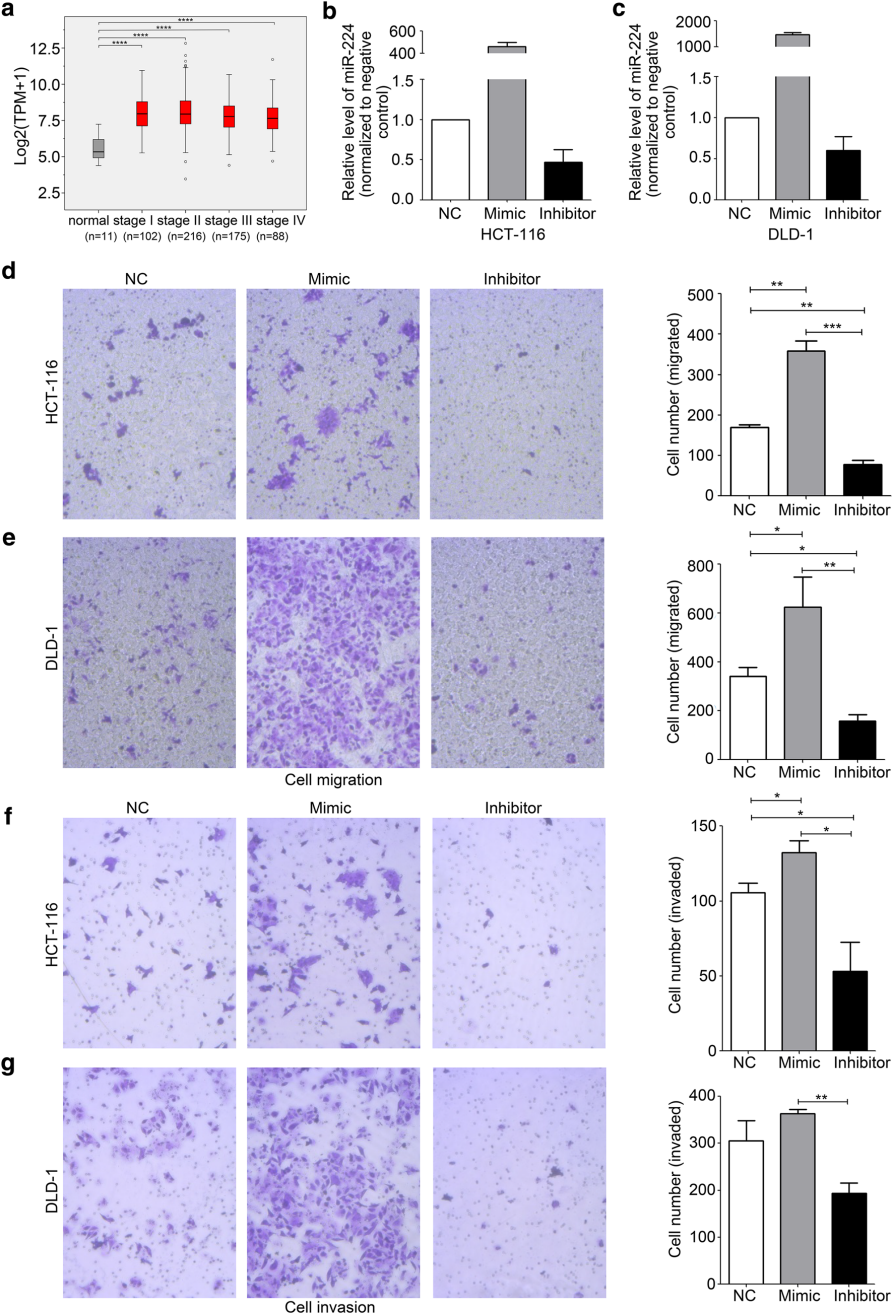
miR-224 overexpressed in CRC tissues promotes cell migration and invasion of human CRC cells. **a** miR-224 expression was increased in CRC tumor tissues in all the stages compared with the normal tissues according to the data from TCGA, but miR-224 levels had no significant differences statistically in comparison between any two stages. TPM: transcripts per million. ****p < 0.0001. **b** Gene expressions of miR-224 after 48 h of transfection with miR-224 mimics or miR-224 inhibitors in HCT-116 cells. **c** Gene expressions of miR-224 after 48 h of transfection with miR-224 mimics or miR-224 inhibitors in DLD-1 cells by RT-qPCR. The expression of miR-224 was normalized by miR-224 expression in NC-transfected cells. NC: negative control. **d**, **e** Representative images (left) and quantification (right) of migrated cells across a transwell insert in HCT-116 and DLD-1 cells after 48 h of transfection with miR-224 mimics or miR-224 inhibitors, respectively. *p < 0.05, **p < 0.01, ***p < 0.001. **f**, **g** Representative images (left) and quantification (right) of invaded cells across a transwell insert in HCT-116 and DLD-1 cells after 48 h of transfection with miR-224 mimics or miR-224 inhibitors, respectively. *p < 0.05, **p < 0.01

To investigate the miR-224 expression in CRC, we analyzed the data from TCGA which showed a high expression level of miR-224 in all stages of CRC compared with the normal tissues (****p < 0.001, Fig. 1). Additionally, miR-224 level was higher in COAD than in READ but had no significant difference in genders, CRC stages, or T-classified subgroups based on TNM classification (Supplementary Fig. 1). We further measured the endogenous miR-224 expression in CRC cell lines (HCT-116, DLD-1, and SW-480) together with miR-224 level of a non-CRC cell line (HEK293T) as a control by RT-qPCR. We found miR-224 level was relatively higher in CRC cells, particularly in HCT-116 and DLD-1, compared with HEK293T cells (Supplementary Fig. 2), thus we chose HCT-116 and DLD-1 cells to perform the following experiments.

Since the expression of miR-224 was elevated in CRC tissues and cell lines, we explored its role in CRC cells to better understand its molecular mechanism during CRC development. First, we transfected miR-224 mimic, inhibitor and negative control (NC) to increase or decrease the miR-224 levels in HCT-116 and DLD-1 cells (Fig. 1), and then conducted transwell assays which showed that transfection of miR-224 mimic significantly increased migration ability in both HCT-116 (*p *< 0.01) and DLD-1 (*p *< 0.05), while miR-224 inhibitor suppressed cell migration in HCT-116 (*p *< 0.01) and DLD-1 (*p *< 0.05) compared to NC (Fig. 1). For cell invasion, the penetrated cell number also significantly rose after transfection of miR-224 mimic (*p *< 0.05) but was reduced by miR-224 inhibitor (*p *< 0.05) in comparison to NC in HCT-116 cells (Fig. 1). For cell invasion in DLD-1 cell lines, it carried an identical trend though there was no statistical significance (Fig. 1).

### miR-224 binds to its target *BTRC* and controls *BTRC* expression

**Fig. 2:**
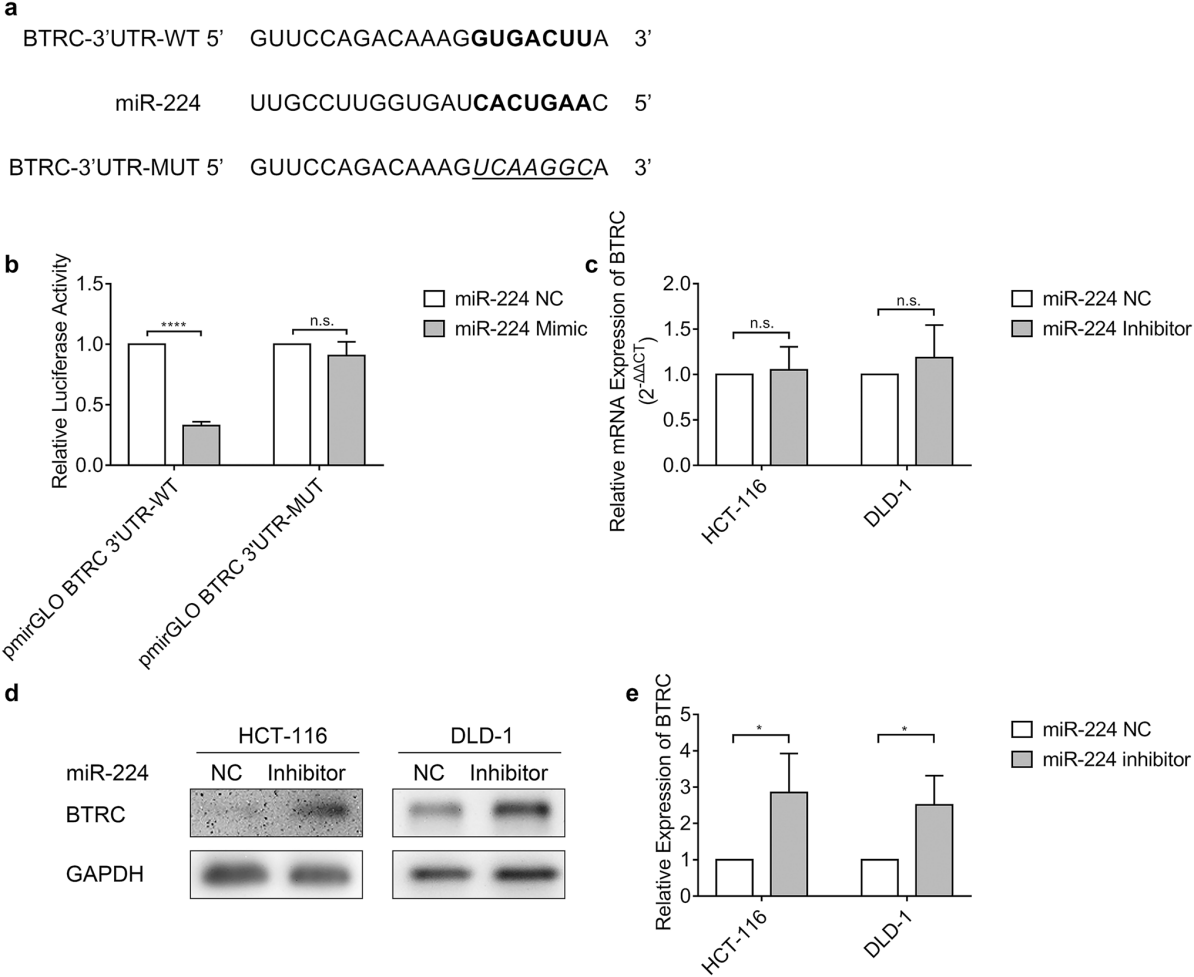
miR-224 targets *BTRC* and regulates its expression in protein level but not in mRNA level. **a** MiRecords predicted that there existed a seed region with the complementary base pairing (**bold**) between miR-224 and the 3′UTRs of *BTRC*, and this seed region in pmirGLO BTRC 3′UTR-WT was mutated into the sequences (*Italic with underline*) to reconstruct the plasmid of pmirGLO BTRC 3′UTR-MUT. WT: wild type, MUT: mutant. **b** The dual-luciferase activity of pmirGLO-BTRC-3′UTR-WT and pmirGLO-BTRC-3′UTR-MUT in HEK293T cells after co-transfection with miR-224 mimic or NC showed the interaction between miR-224 and 3′UTR of *BTRC*. ****p < 0.0001, n.s. = no significance. **c** The expression of *BTRC* in HCT-116 and DLD-1 cells transfected with miR-224 inhibitor or negative control (NC) in mRNA level by RT-qPCR, normalized to negative controls. GAPDH was used as an endogenous control. n.s. = no significance. **d** Representative images (left) and the relative quantification (right) of the protein expression levels of BTRC in HCT-116 and DLD-1 cells transfected with miR-224 inhibitor or negative control (NC), normalized to negative controls by western blot. Western blot bands were quantified by ImageJ and GAPDH was used as an endogenous control. *p < 0.05

To understand the molecular mechanism on how miR-224 promoted cell migration and invasion, we used miRecords and KEGG database to select predicted target genes in cancer-related pathways and then found that *BTRC* in the Wnt/β-catenin pathway with a putative miR-224 binding site (Fig. 2) might be a potential target of miR-224. To confirm that miR-224 could bind to the *BTRC* mRNA 3′UTR, we constructed wild-type (pmirGLO-BTRC-3′UTR-WT) and mutant (pmirGLO-BTRC-3′UTR-MUT) plasmid containing the sequence of the 3′UTR of *BTRC* including the predicted miR-224 target site (Supplementary Table 1), and then performed dual-luciferase reporter assay. The luciferase activity of pmirGLO-BTRC-3′UTR-WT saw a significant fall in HEK293T cells after transfection of miR-224 mimic compared to NC (*p *< 0.0001, Fig. 2), but that of pmirGLO-BTRC-3′UTR-MUT with artificial mutant seed region (Fig. 2) remained little changed between miR-224 mimic and NC-transfected HEK293T cells (Fig. 2).

Furthermore, we conducted qRT-PCR and western blot to determine the effects of miR-224 on *BTRC* expression. The results showed that there was no significant difference in *BTRC* mRNA level between miR-224 inhibitor-transfected cells and NC-transfected cells in both HCT-116 and DLD-1 (Fig. 2). However, the protein expression of *BTRC* was increased in HCT-116 (*p *< 0.05) and DLD-1 (*p *< 0.05) after transfection of miR-224 inhibitor compared with NC (Fig. 2). These data revealed that miR-224 can mediate *BTRC* expression in protein level rather than in mRNA level.

### Inhibition of miR-224 represses *BTRC*-related Wnt/β-catenin pathway

**Fig. 3:**
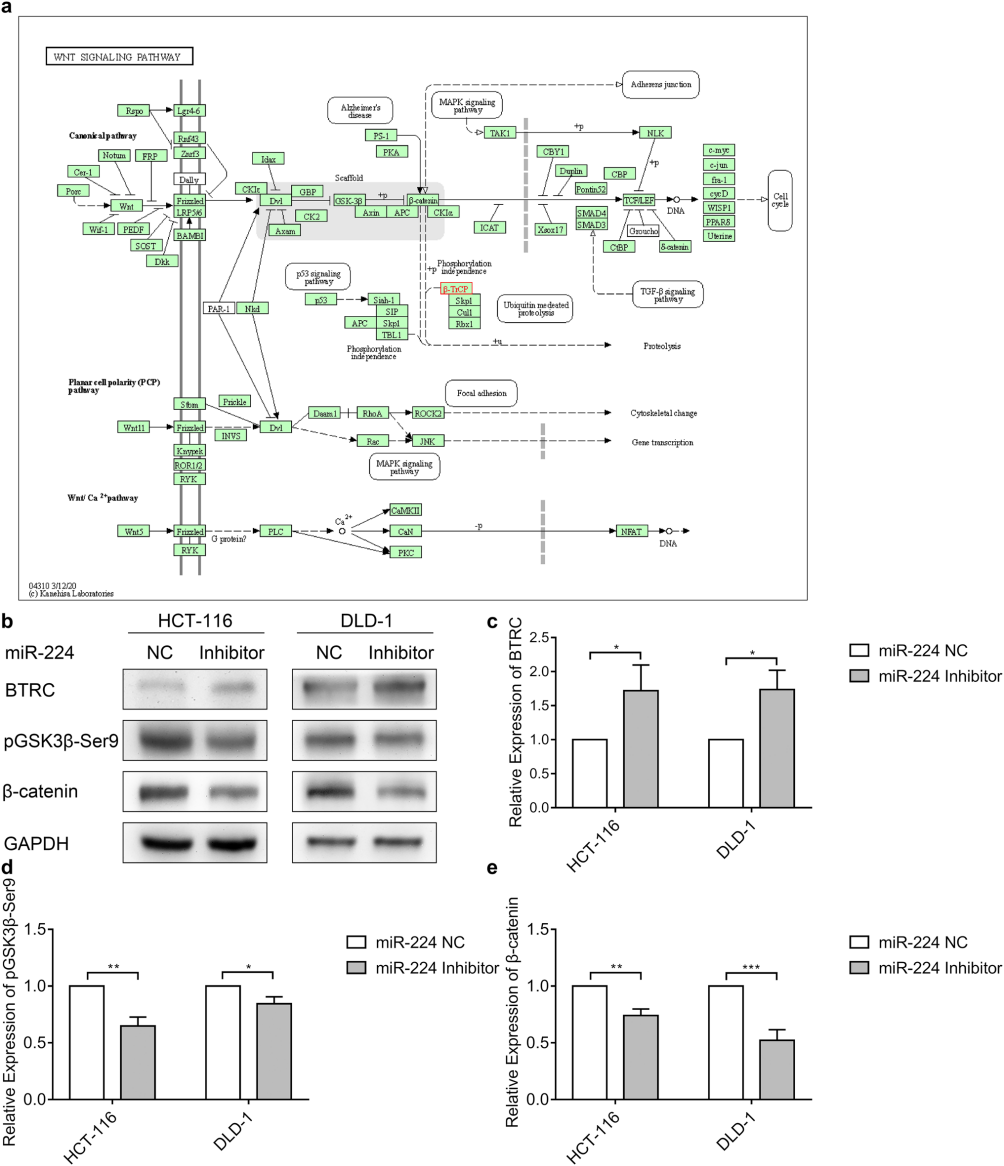
miR-224 represses *BTRC*-related Wnt/β-catenin pathway. **a** The position of *BTRC* in Wnt pathway in KEGG pathway map. β-TrCP is an alias of *BTRC*. **b** The protein expression of BTRC, pGSK3β-Ser9, and β-catenin in HCT-116 and DLD-1 cells after 48 h transfection with miR-224 inhibitor and negative control (NC) determined by western blot. GAPDH was used as an endogenous control. **c–e** The quantification of the protein expression levels of BTRC, pGSK3β-Ser9, and β-catenin, respectively, normalized to negative controls. Western blot bands were quantified by ImageJ and GAPDH was used as an endogenous control. *p < 0.05, **p < 0.01. ***p<0.001

The role of *BTRC* in the Wnt pathway is to recruit and then to degrade cytoplastic phosphorylated β-catenin in a ubiquitin-dependent way (Fig. 3), resulting in reduction of accumulation of nuclear β-catenin which is highly associated with metastasis (Su et al. 2008; Zhou et al. 2017). Our previous study also demonstrated that reduced expressions of pGSK3β-Ser9 and β-catenin in CRC cells were consistent with lower cell migratory and invasive abilities (Zhou et al. 2017), thus we hypothesized that miR-224 inhibition would affect the Wnt/β-catenin pathway as well. Compared with negative controls in both cell lines, when the *BTRC* expression level was rescued by transfection of miR-224 inhibitor, we did observe that the expressions of both pGSK3β-Ser9 and β-catenin dropped (Fig. 3).

### BTRC protein expression in CRC tissue is downregulated

**Fig. 4:**
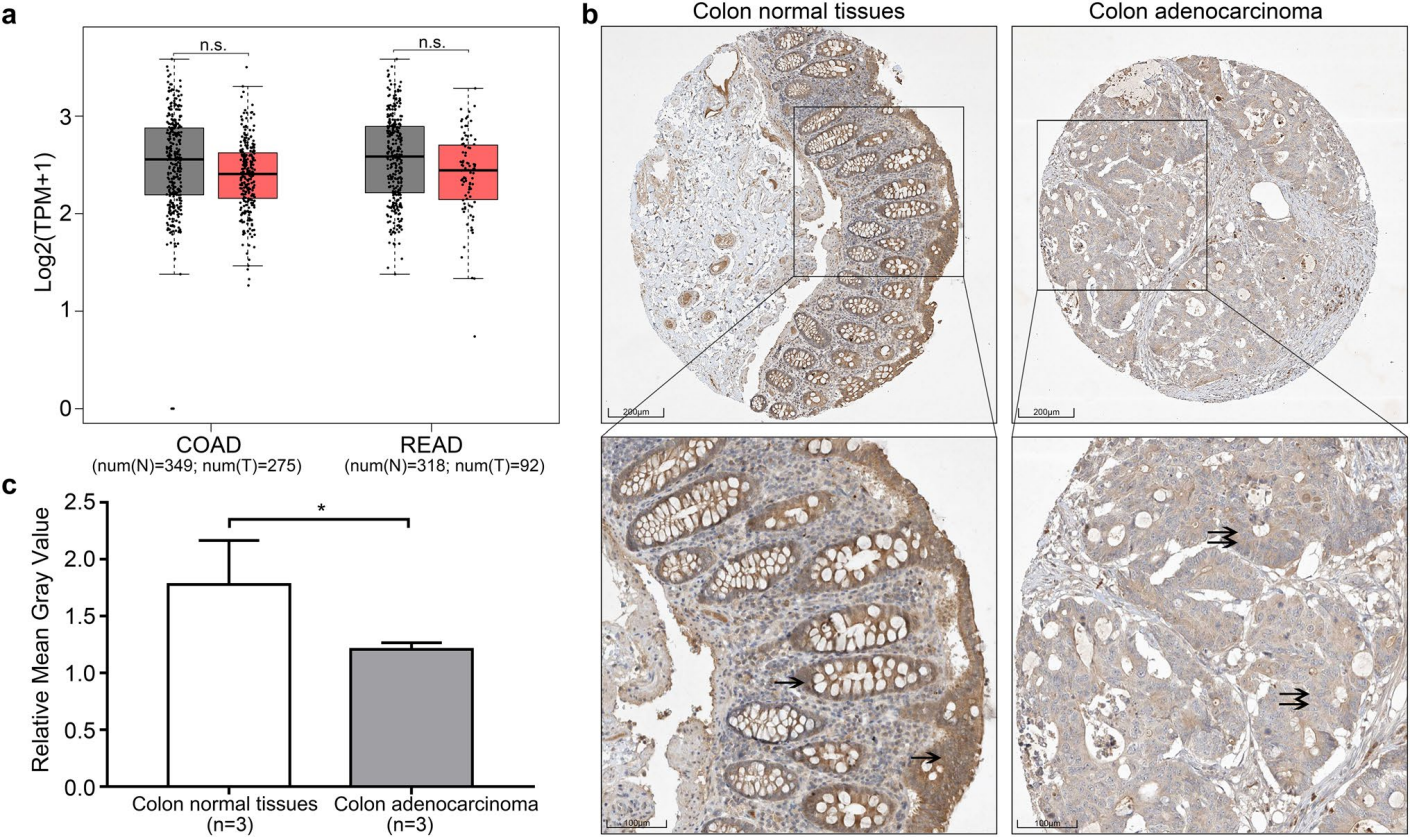
The positive expression of *BTRC* is stronger in CRC than in normal tissues. **a** *BTRC* transcripts in normal tissues (N) and tumor tissues (T) of READ and COAD from TCGA data. TPM: transcripts per million. n.s. = no significance**. b** The representative IHC pictures of BTRC protein expression in colon normal tissues and CRC tissues by the Human Protein Atlas. Scale bar is 200 μm (upper) and 100 μm (lower). The single arrowheads point to the positive intensity of BTRC in normal colon tissues, and the double arrowheads point to the positive intensity of BTRC in colon adenocarcinoma. BTRC expression in colon adenocarcinoma was lower than in colon normal tissues. **c** ImageJ was used to analyze the relative mean gray values of BTRC protein expression in IHC pictures of colon normal tissues (n = 3) and colon adenocarcinoma (n = 3) which were normalized to the corresponding mean gray values of stromal tissues in each IHC picture from the Human Protein Atlas. Relative gray value = (The intensity of BTRC expression in normal colorectal epithelium or colorectal tumor)/The intensity of BTRC expression in colorectal stromal cells. *p < 0.05

To further compare the *BTRC* expression in CRC and normal colorectal epithelial tissues, we first analyzed *BTRC* transcripts in COAD and READ based on data from TCGA via GEPIA and both had no significant difference between normal tissues and carcinoma tissues (Fig. 4). Then, we referred to the IHC pictures in Human Protein Atlas and found that the protein expression of BTRC was lower in CRC than in normal colorectal epithelium (Fig. 4). Furthermore, we used ImageJ to evaluate BTRC protein expression by quantitative analysis of the relative gray value, which showed a reverse trend to the miR-224 expression level in CRC (*p *< 0.05, Fig. 4).

## Discussion

CRC is still the fourth most common cancer across the world and the most important cause of death is metastasis. Effective biomarkers to identify high metastatic risk CRC patients at early stage or to serve as a potential therapeutic target are needed to promptly and effectively treat this group of patients to improve the overall outcome for CRC patients. In this study, we report that elevated miR-224 expression level is found in CRC tissues and cell lines and inhibition of miR-224 could upregulate the protein expression of targeted *BTRC*, suppress the Wnt/β-catenin pathway, and reduce the migratory and invasive abilities of CRC cells. The protein expression, but not transcripts, of *BTRC* was downregulated in CRC tissues compared to normal tissues based on TCGA and the IHC data from the Human Protein Atlas.

We proved that miR-224 is an onco-miRNA rather than suppressor. The role of miR-224 differs in various types of cancer (Miao et al. 2018; Zhang et al. 2019) and it is also controversial in CRC. Ling and their colleagues reported that miR-224 was an onco-miRNA in CRC and expression of miR-224 was higher at advanced stages of CRC in most datasets (Ling et al. 2016) while another study demonstrated that miR-224 was decreased in CRC compared to normal tissues and suppressed the migration of CRC cells (Ke et al. 2014). Compared to the latter, the former studies with a larger scale of clinical specimens and several other findings (Adamopoulos et al. 2015; Liao et al. 2013) presented more solid evidence that miR-224 served as an onco-miRNA. These different results of miR-224’s role in CRC might be attributed to small sample sizes, or heterogeneity in CRC patients and cell lines due to multiple mutation status of CRC. Here, we prove that miR-224 level is higher in CRC than in normal tissues. Additionally, we further determined that the miR-224 expression was also higher in COAD than in READ, but its molecular mechanism needs to be further studied. On the other hand, we found that overexpression of miR-224 can promote cell migration and invasion in primary CRC cell lines HCT-116 and DLD-1, while inhibition of miR-224 has a reverse effect. As the expression of miR-224 is elevated in stage I of CRC and does not increase in the advanced stages according to the TCGA analysis, these results suggest that miR-224 can promote cell migration and invasion but may not be related to CRC metastasis, supporting that miR-224 might be a promising biomarker or target for early-stage non-metastatic CRC treatments.

For in vitro study, heterogeneity of cancer cell lines might account for the lack of significance in cell invasion of DLD-1 cells after transfection of miR-224 mimic and negative control, respectively. In effect, we found that the endogenous miR-224 expressions in HCT-116 and DLD-1 cells were relatively high so transfection with miR-224 mimics might be redundant and thus had little impact on downstream functions and signaling pathways. This is also the reason why we only used miR-224 inhibitor for miR-224 target *BTRC* and downstream pathway.

We demonstrate that *BTRC* is a novel target of miR-224 and miR-224 can inhibit its protein level but not mRNA level in HCT-116 and DLD-1 cells. The dual-luciferase assay of wild-type and mutant plasmid containing the 3′UTR sequence of *BTRC* indicated that the GUGACUU on 3′UTR of *BTRC* (bold in Fig. 2) was the binding site of miR-224. It suggests that miR-224 regulates *BTRC* through translational repression. In addition, in the context of an overexpression of miR-224 in CRC, the *BTRC* transcripts in CRC are not significantly changed while the protein expression of BTRC would be reduced in CRC tissues in comparison to normal colorectal tissues according to the IHC from the Human Protein Atlas. These data were consistent with our results in CRC cells. However, we think that the correlation between BTRC protein expression and CRC patients still needs further validation on a larger scale.

In addition, our study shows that Wnt/β-catenin signaling can be suppressed when the protein expression of BTRC is rescued by miR-224 inhibitor. *BTRC* encodes a member of the F-box protein family which functions in phosphorylation-dependent ubiquitination and it is an essential part of ubiquitin-mediated proteolysis of β-catenin in the Wnt pathway (Clevers and Nusse 2012; Li et al. 2012). A series of pathological Wnt signaling activation is a classic hallmark of CRC and β-catenin is the key intracellular signal transducer in the canonical Wnt pathway (Nusse and Clevers 2017). In the absence of Wnt ligands, cytoplasmic β-catenin can be phosphorylated by a multi-protein destruction complex composed of Adenomatous polyposis coli (APC), Axin-like protein (Axin 1/2), Glycogen synthase kinase (GSK3β) and Casein kinase (CK1α) (Kleeman et al. 2019) and then recruited and degraded by BTRC. When β-catenin degradation is blocked, accumulated β-catenin in cytoplasm will translocate to the nucleus, activating a large number of Wnt target genes (Kleeman et al. 2019), which implies that upregulation of BTRC protein might activate Wnt signaling leading to promotion of cell migration and invasion abilities. It is known that Wnt signaling plays an essential part in the early stage of CRC, but in this study, we address that miR-224 might mediate BTRC expression and control cell migration and invasion by interfering Wnt pathway, which is analogous to our previous study that the Wnt/β-catenin pathway mediated by hsa-miR-650 could affect CRC cell migration and invasion (Zhou et al. 2017). In that study, we also proved that the reduced expression of pGSK3β-ser9 eliminated the repression of GSK3β activity and the decrease in β-catenin protein level was correlated with an inhibited aggressive phenotype (Zhou et al. 2017). These data exhibit that miR-224 might contribute to the metastasis in the early stage of CRC through the Wnt pathway, indicating that it has the possibility to be a novel biomarker or target for the therapies of high-risk non-metastatic CRC.

Furthermore, it has been reported that some important tumor suppressor genes such as *PTEN* (Yang et al. 2018) and *SMAD4* (Cheng et al. 2018) are also targets of miR-224, which does not contradict our idea that miR-224 can control CRC progression partly through *BTRC*-mediated Wnt/β-catenin signaling pathway. The characteristic that miRNAs can target multiple mRNAs altered in pathological conditions is the advantage of miRNAs as the targets of therapeutics(Rupaimoole and Slack 2017). During the development of CRC, a variety of changes enhances its carcinogenesis. Thus, we hypothesize that miR-224 targeting *BTRC* can amplify its impact on alteration in the aggressive phenotype of CRC cells, and we believe that miR-224 is a prospective therapeutic target for CRC treatment. In particular, we think it has a great potential to be a biomarker or treatment target for high-risk non-metastatic CRC patients. However, we must admit that whether negative regulation of *BTRC* by miR-224 can control Wnt/β-catenin pathway and phenotypic alteration independently still needs further exploration.

In summary, we have identified that miR-224 has an increase in its expression in CRC tumor tissues in comparison to normal tissues and can promote CRC cell migration and invasion. Inhibition of miR-224 could increase BTRC protein expression and repress Wnt/β-catenin signaling, leading to a decline in cell migration and invasion abilities. BTRC protein expression might be reduced in CRC tissues compared to normal colorectal epithelium. Therefore, we believe that inhibition of miR-224 might be a potential therapeutic strategy, especially for those early-stage CRC patients with high risk of metastasis.

## Electronic supplementary material

Below is the link to the electronic supplementary material.

Supplementary material 1 (DOCX 303 kb)
