# Supplementary material for: miR-224 targets BTRC and promotes cell migration and invasion in colorectal cancer
Source: 3 Biotech. 2020 Oct 24;10(11):485. doi: 10.1007/s13205-020-02477-x (PMC7585582; doi:10.1007/s13205-020-02477-x)
Supplement: Supplementary file 1 — Supplementary material 1 (DOCX 303 kb) [file 13205_2020_2477_MOESM1_ESM.docx]

**Supplementary Table.1**

The sequences inserted in the pmirGLO to reconstruct BTRC 3’UTR-WT or BTRC 3’UTR-MUT plasmid

| Names of inserts | Inserted sequences (5’→3’) |
| --- | --- |
| BTRC 3’UTR-WT | *GTTTAAAC*TCCTTTCACCTCTGCACCTAGTTTTTTCCCATTGGTTCCAGACAAAG**GTGACTT**ATAAATATATTTAGTGTTTTGCCAGAATCTCTCT*TCTAGA* |
| BTRC 3’UTR-MUT | *GTTTAAAC*TCCTTTCACCTCTGCACCTAGTTTTTTCCCATTGGTTCCAGACAAAG**TCAAGGC**ATAAATATATTTAGTGTTTTGCCAGAATCTCTCT*TCTAGA* |

Notes: restriction sites are in *Italic*; wild-type or mutant seed regions are in **bold**.

**Supplementary Table.2**

The primers used for measurement of BTRC mRNA levels. GAPDH was used as an endogenous control

| Primes | Sequences (5’→3’) |
| --- | --- |
| GAPDH-Forward | CTGACTTCAACAGCGACACC |
| GAPDH-Reverse | TGCTGTAGCCAAATTCGTTGT |
| BTRC-Forward | CCAGACTCTGCTTAAACCAAGAA |
| BTRC-Reverse | GGGCACAATCATACTGGAAGTG |

**Supplementary Fig.1**


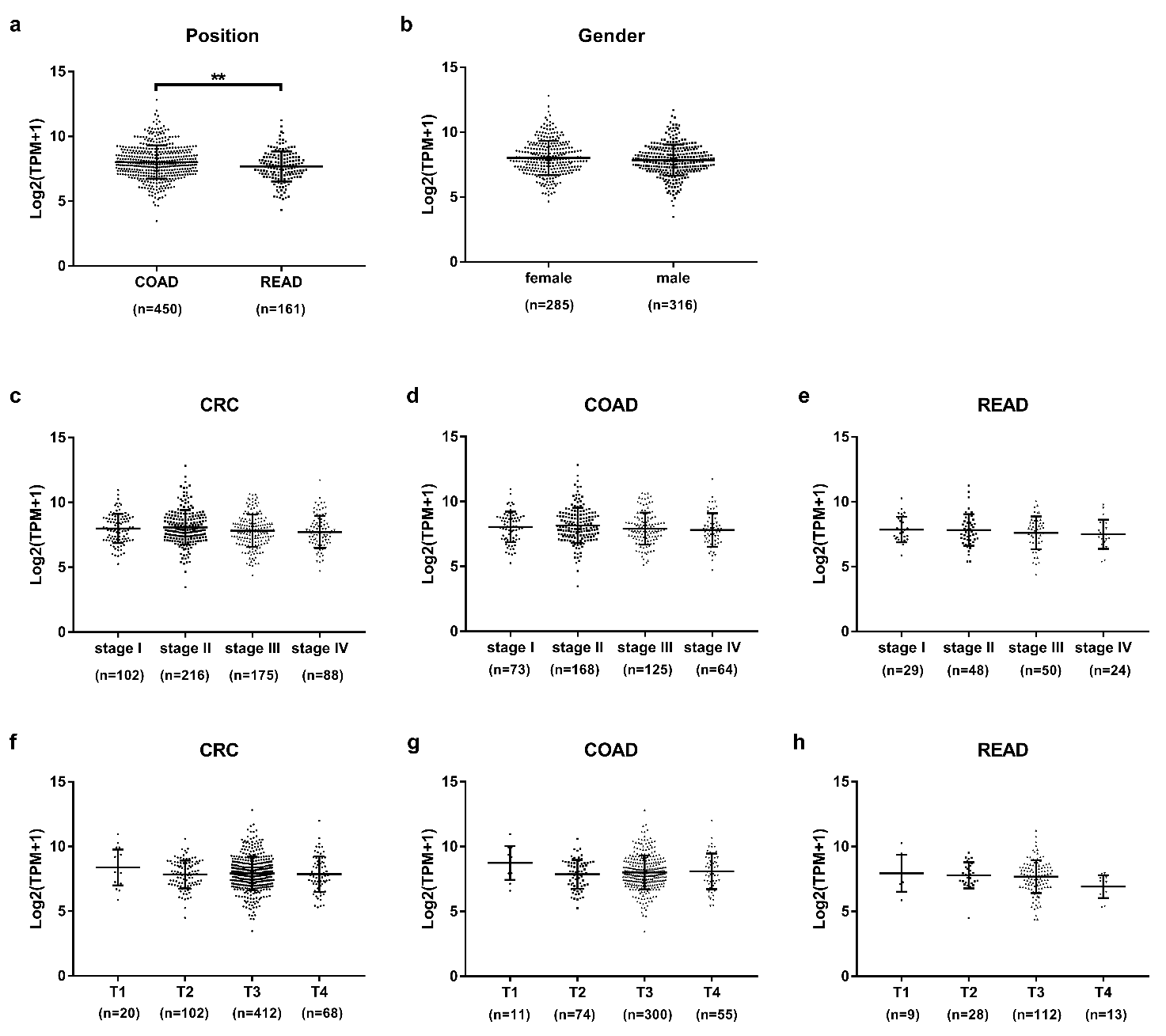


**Supplementary Fig.1** Analyses of miR-224 level in different CRC position, genders, stages and T subgroups. **a** miR-224 level was higher in COAD than in READ. *p***<0.01. **b** There was no significant difference between miR-224 levels in female and male CRC patients. **c-e** There was no significant difference among miR-224 levels in different stages of CRC, COAD, or READ. **f-h** There was no significant difference among miR-224 levels in different T subgroups of CRC, COAD, or READ

**Supplementary Fig.2**


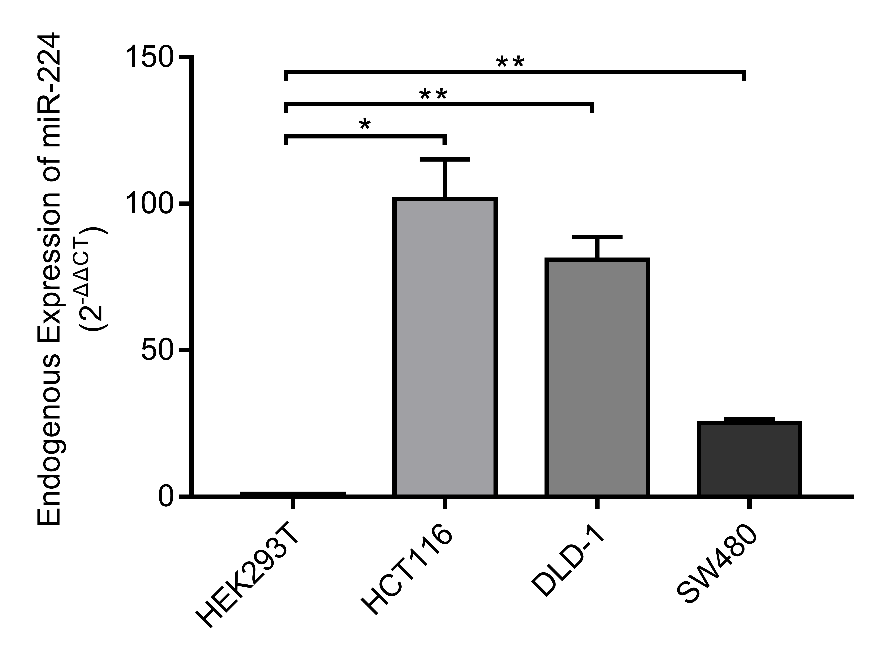


**Supplementary Fig.2** The endogenous expression of miR-224 in CRC cell lines (HCT-116, DLD-1, SW480) was higher than in 293T cells by RT-qPCR. MiR-224 expression were normalized to mR-224 level in 293T cells. U47 was used as an endogenous control. *p**<0.05, *p***<0.01
